# Interaction of soil pH, organic matter, exchangeable acidity, and cation exchange capacity in a managed tea farm

**DOI:** 10.7717/peerj.20341

**Published:** 2025-11-24

**Authors:** Haijie Song, Jing Shi, Rou Wang, Tao Jin, Yishu Peng

**Affiliations:** 1 College of Tea Science, Guizhou University, Guiyang, Guizhou Province, China; 2 Institute of Mountain Resources of Guizhou Province, Institute of Mountain Resources of Guizhou Province, Guiyang, China

**Keywords:** Land use type, Managed tea garden, Soil acidification, Soil profile, Surface soil, Unmanaged tea garden

## Abstract

**Background:**

The Yangai tea farm was established in 1952, with a long history of cultivating tea plants. The tea plant can activate Al^3+^ of the soil and affect soil physicochemical properties. Understanding soil physicochemical properties is crucial for studying soil fertility, nutrient retention, and long-term agricultural sustainability. Therefore, we investigated the distribution characteristics of soil pH, exchangeable acid, soil organic matter (SOM), and cation exchange capacity (CEC) in the surface soil and soil profile to assess their interaction in the managed tea garden.

**Methods:**

We collected surface soil and soil profile samples from the managed tea garden and three land-use types (*i.e.*, managed tea garden, unmanaged tea garden, and *Pinus massoniana* forest), respectively. We measure soil pH value with a laboratory pH meter. We measured the SOM, soil exchangeable acid, and CEC content, respectively, with potassium dichromate heating, potassium chloride leaching, and spectrophotometric methods.

**Results:**

The average soil pH value, exchangeable Al^3+^ content, and exchangeable H^+^ content were 4.50 (ranging from 3.95 to 5.88), 6.11 (0.04 to 9.32) cmol kg^−1^, and 0.30 (0.03 to 0.62) cmol kg^−1^, respectively. The surface soil acidification is severe, and the exchangeable acids in the tea garden were mainly exchangeable Al^3+^. The surface soil of the Yangai tea farm had a high ability to keep and supply fertilizer due to its enriching SOM content (average 55.94g kg^−1^) and soil CEC (24.06 cmol(+) kg^−1^). Surface soil exchangeable acid and CEC contents were higher after spring tea picking than before spring tea picking in the Yangai tea farm, while their SOM content was just the opposite. Therefore, it was recommended to supplement organic fertilizers after tea-picking because the surface SOM of the Yangai tea farm would decompose and be consumed more during the spring, summer, and autumn tea-picking periods than in winter. In addition, the SOM contents and soil pH values decreased and then increased with the increasing soil depth of the soil profile in three land-use types. The soil acidification rate of the managed tea garden was faster than that of the unmanaged tea garden and *Pinus massoniana* forest, and the difference in the acidification rate between the unmanaged tea garden and *Pinus massoniana* forest was slight. Therefore, there should be attention to preventing excessive soil acidification in the later tea garden management.

## Introduction

Soil pH, exchangeable acid, soil organic matter (SOM), and cation exchange capacity (CEC) are important factors affecting soil fertility and productivity, soil structure, nutrient retention, and microbial activity, contributing to plant health growth and increasing crop yield. SOM maintains many ecosystem functions, such as enhancing soil water capacity (Lal, 2020), preventing land drought, and acting as an energy and nutrient source for soil microorganisms (Hoffland et al., 2020). Soil CEC refers to the total amount of various cations adsorbed by soil colloids, including acid-causing ions (such as H^+^ and Al^3+^) and basic cations (*i.e.,* K^+^, Na^+^, Ca^2+^, Mg^2+^, *etc*.), which is widely used as an indicator of soil nutrient availability and buffering capacity (Solly et al., 2020). Soil pH and exchangeable acid (*i.e.,* exchangeable H^+^ and Al^3+^) are indicators of soil acidification. Tea plantations are particularly susceptible to soil acidification, which can lead to reduced nutrient availability and increased risks of aluminum toxicity, inhibiting root elongation and nutrient absorption and ultimately affecting tea plant health and yield. Soil acidification can cause farmland aluminum toxicity, one of the most growth-limiting factors in acidic soil (Zeng et al., 2017; Zhu et al., 2018).

Soil pH, exchangeable acid, SOM, and CEC were affected by tea planting. The tea plant can activate Al^3+^ in the soil and accumulate in the old leaves (Peng et al., 2022), resulting in pruned tea leaves that may further accelerate soil acidification (Yan et al., 2018). Long-term tea planting will also cause changes in the soil physicochemical properties (such as soil pH, exchangeable acid, SOM, and CEC) (Wang, Li & Zheng, 2018). Soil physicochemical properties are different with dissimilar land use types (Barbero et al., 2025). In addition, as a complex ecosystem, soil physicochemical properties interact. Soil pH of the woodland and grassland soil profiles in the Lancangjiang River Basin in southwest China increased with soil depth and negatively correlated with organic carbon content (Zhou et al., 2020). Soil pH positively correlated with exchangeable Al^3+^, exchangeable Ca^2+^, and annual crop yield under the wheat-corn planting model in Qiyang County, South China (Qaswar et al., 2020). Therefore, plant planting species and time can affect soil physicochemical properties.

The Yangai tea farm, a long history of cultivating tea plants, established in 1952, is located on a hilly platform in the southwest of Guiyang City, Guizhou Province, China. As soil acidification is a primary constraint in tea plantations, understanding the spatial distribution and interactions of key soil physicochemical properties is essential for developing targeted soil management strategies. Therefore, this study measured and analyzed the soil pH, exchangeable acid, SOM, and soil CEC in the surface soil at different periods and the soil profiles of three soil use types, to understand the interaction of various soil physicochemical properties and to provide a scientific theoretical basis for green sustainability management of the Yangai tea farm.

## Materials & Methods

### Study area

The Yangai tea farm, on gentle slopes on high mountain terraces and diverse microtopography, built in 1952, is located at 26°23′19″N and 106°31′27″E, covering an area of more than 1,333 hectares. The average altitude of the Yangai tea farm is 1,300 m, and the climate type belongs to a subtropical humid climate. The annual rainfall in the research area is 1,125 mm, the frost-free period is about 247 days, the average yearly temperature is 14.2 °C, the annual total accumulated temperature is 4,300 °C, the temperature difference between day and night is 3∼13 °C, and there are about 290 rainy days a year, and the air is humid. The soil of the Yangai tea farm is Acrisols (FAO), and its tea plant variety is the early bud and medium leaf group of *Camella sinensis* (L) O. Ktze. Cv. Kunming Shilixiang in northern Yunnan, which germinates earlier and has denser hairs on the back of leaves than the local varieties, and the taste is fresher. At present, the Yangai tea farm mainly applies organic fertilizers. Therefore, there are more clouds and fog in the early spring of the Yangai tea farm, and the air is humid, which provides good external conditions for the tenderness and excellent quality of tea buds.

### Sample collection

**Figure 1:**
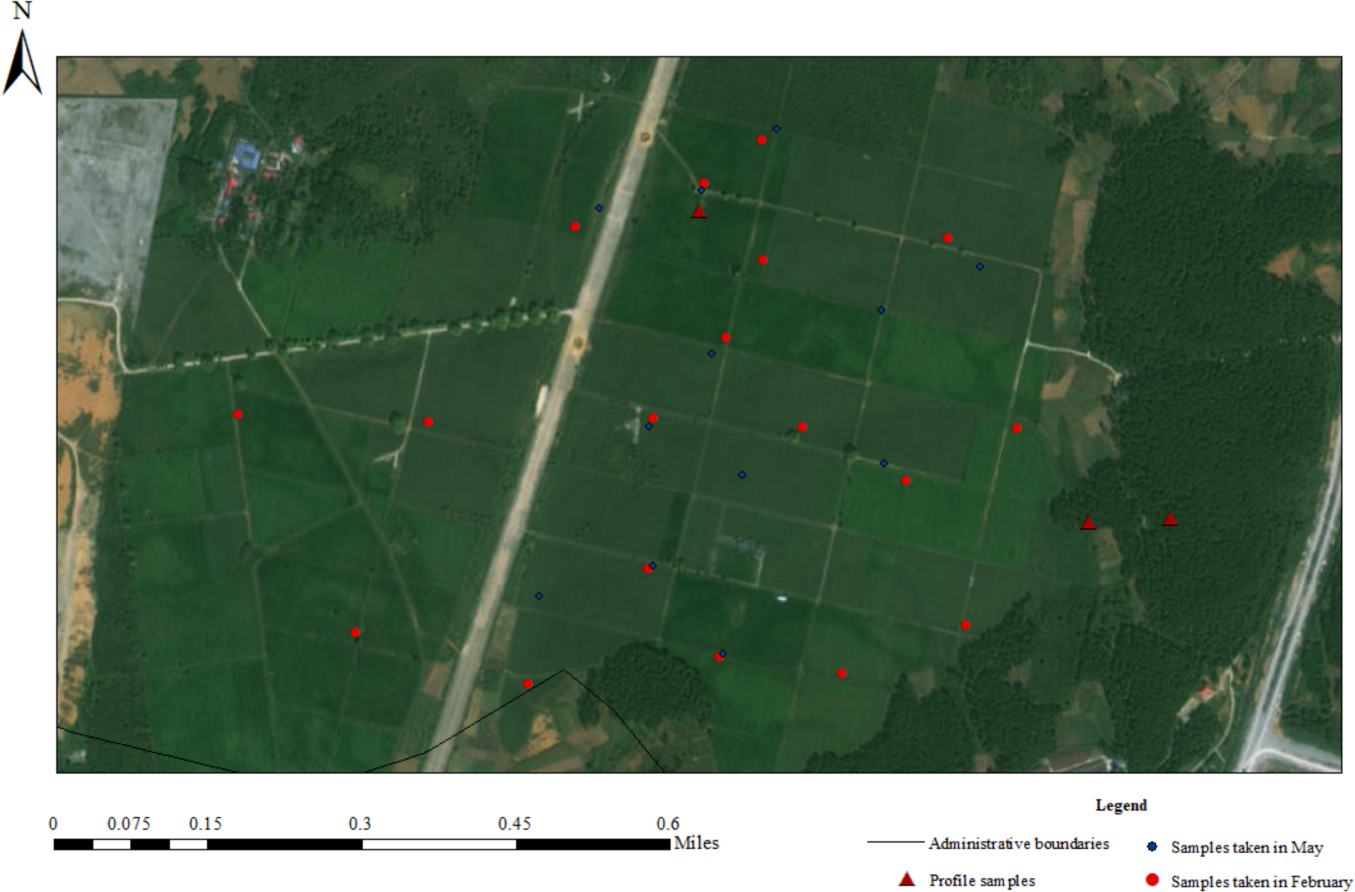
Sampling location of the research area.

To understand whether spring tea picking will affect soil physicochemical properties in the tea garden, this study collected 18 and 12 surface soil samples in February and late May 2023 from the Yangai tea farm in Guiyang City, Guizhou Province, respectively. The surface soil sample was collected and mixed from five soil samples of different rows in the tea garden around the collected point at a soil depth of 5∼30 cm. In addition, we also collected three soil profiles of various land use types (*i.e.,* *Pinus massoniana* forest soil, unmanaged tea garden soil, and managed tea garden soil) (Fig. S1) in February 2023. Through observation, the profile soil is sampled by the “X” method after determining the midpoint, and five sub-samples collected in each layer are mixed into one sample respectively, a total of 19 soil samples. The topsoil sample of profile at a soil depth of 0∼10 cm and additional samples were collected at 20-cm intervals below the surface soil layer to ensure adequate vertical resolution for our analyses. We collected seven samples from the soil section in the managed tea garden with a sampling soil depth of 130 cm and six samples from other soil profiles with a sampling soil depth of 110 cm. The sampling locations in this study are presented in Fig. 1 and Table S1.

Each soil sample was about one kg. We packed soil samples using a self-sealing bag, brought them back to the lab, dried them in a constant temperature drying box at 30 °C, and passed through a two mm nylon sieve to remove coarse particles, ensuring uniform sample preparation for subsequent analyses. We ground them and then screened them with nylon screens for reserve use according to the experimental requirements. The comprehensive workflow of the experimental procedure is shown in Fig. S2.

### Soil pH value measurement

We weighed 10.00 g of the dried soil sample, put it into a 50 mL small beaker, and added 25 mL of deionized water. The mixture was stirred with a glass rod for one minute and allowed to stand for 30 min. We measured the soil pH values with a pH meter (PHS320, Chengdu Century Ark Technology Co., LTD., Chengdu, China). Before measuring, the pH meter was calibrated with standard buffer solutions.

### Soil exchangeable acid content determination

The soil exchangeable acid was measured using the potassium chloride leaching method (Yan et al., 2020). The dried soil samples were through a two mm nylon screen, weighed 10 g, and put into a funnel with filter paper. Then, we rinsed the soil sample with 1 mol L^−1^ KCl solution in four increments of 25 mL each (total 100 mL) and put the liquid in a 250 mL volumetric flask to fix the volume. Finally, we put the 200 mL liquid in two 250 mL conical bottles and boiled, respectively, each conical flask 100 ml, at a low temperature for 5 min. One part was titrated directly with 0.02 mol L^−1^ NaOH standard solution with phenolphthalein as an indicator. The other part added one mL of 3.5% NaF solution while hot and titrated with phenolphthalein as an indicator after cooling. The liquids were titrated with 0.02 mol L^−1^ NaOH until it was slightly red and recorded relevant experimental data, the same method for the blank experiment.

### SOM content determination

The SOM content was measured using the potassium dichromate heating method (Lu, 2000). The dried soil samples were through a 0.25 mm nylon screen, weighed 0.1000∼1.000 g, put into a triangular bottle, added 0.4 mol L^−1^ equivalent 1/6 K_2_Cr_2_O_7_ solution 10 mL with a pipette, heated to boiling for 5 min, drop 2∼3 drops of phenanthroline reagent, and then titrate with 0.2 mol L^−1^ ferrous sulfate until brick red, recorded the experimental data.

### Soil CEC determination

We determined the soil CEC using the spectrophotometric method (Feng, Sun & Huang, 2023). In brief, the dried soil samples were passed through a 0.25 mm nylon screen after drying soil samples, weighing 1.75 g, putting them into a 50 mL centrifuge tube, and adding 25 mL 0.0166 M hexamine cobalt chloride solution, tightening the centrifuge tube and placing on an oscillator, adjusting the oscillation frequency at (200 ± 20 rpm) for (60 ± 5) min at (20 ± 2) °C. After the end of the oscillation, the sample was centrifugated at 4,000 rpm for 10 min. The supernatant was carefully filtered into a colorimetric tube and then determined the absorbance by a spectrophotometer at a wavelength of 475 nm and wavelength of 380 nm with 0.0166 M hamming cobalt trichloride solution as a reference.

### Statistical analysis

We used Microsoft Excel 2019 (Microsoft, Redmond, WA, USA) for all the data initially organized and Origin2021 for descriptive statistics, Spearman’s rank correlation analysis, and principal component analysis (PCA). To assess relationships among soil pH, SOM, exchangeable acid, and CEC, we performed Spearman’s rank correlation analysis using Origin 2021. We considered *P* < 0.05 and *P* < 0.01 respective mean statistically significant and highly remarkable. We imported the Excel-organized data into GIS software and conducted spatial interpolation *via* the Kriging method. We choose a spherical model, fix the interpolation parameters, and evaluate the interpolation model accuracy through cross-validation. It ensures the generated soil property distribution maps reflect their spatial variation characteristics.

## Results

### Soil pH distribution characteristics of surface soil in different periods

**Figure 2:**
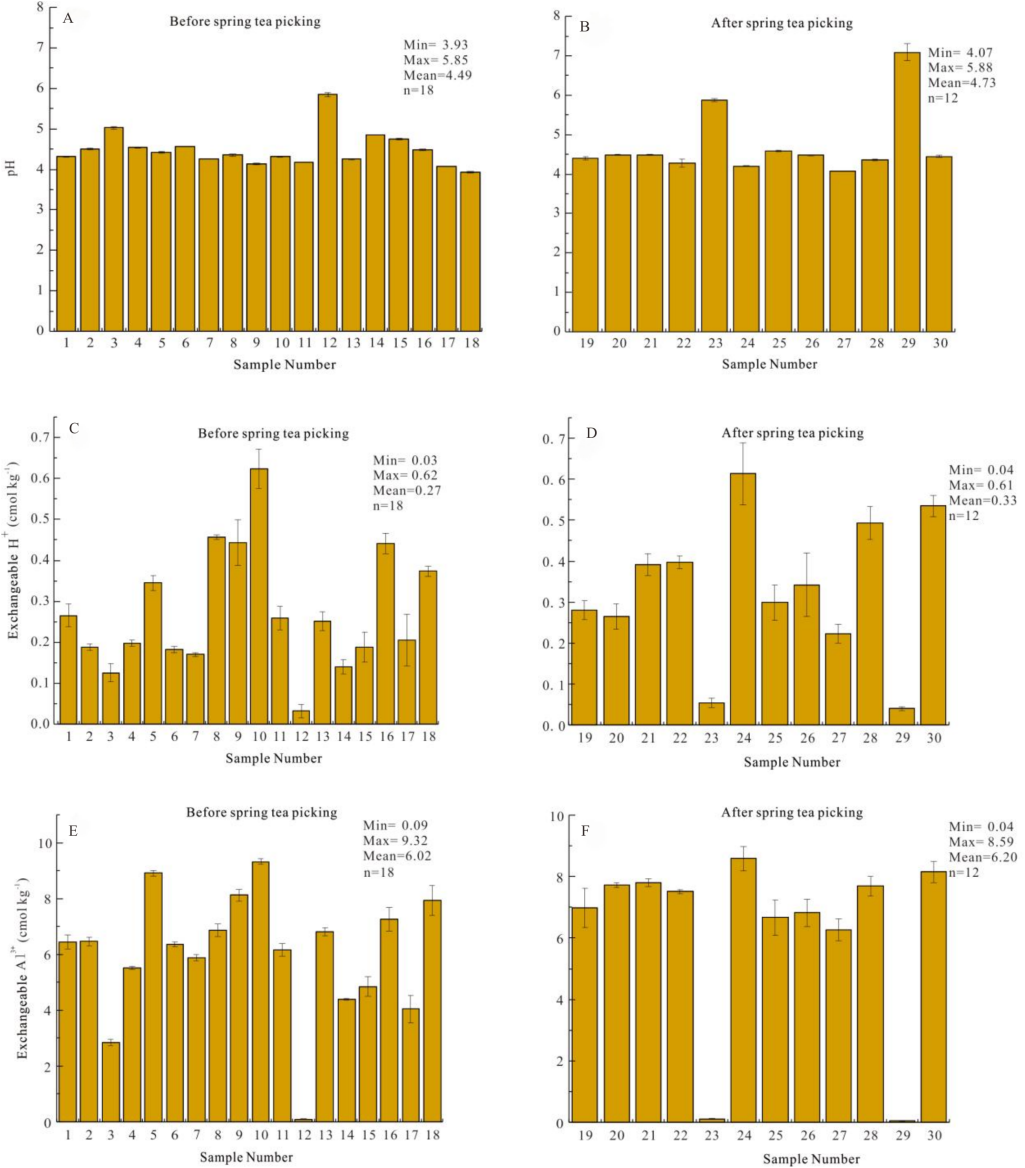
(A) surface soil pH value before spring tea picking; (B) surface soil pH value after spring tea picking; (C) surface soil exchangeable H^+^ contents before spring tea picking; (D) surface soil exchangeable H^+^ contents after spring tea picking; (E) surface soil exchangeable Al^3+^ contents before spring tea picking; (F) surface soil exchangeable Al^3+^ contents after spring tea picking.

The soil pH value of sample No. 29 after spring tea picking was 7.09 and significantly higher than that of other samples in the Yangai tea farm (Fig. 2B). This might be the exposure of some carbonate rocks around the soil sample No. 29 because the soil pH value of carbonate weathering soil is generally high, weakly acidic, or neutral. In the subsequent analysis of soil pH values, we removed it to eliminate this influence. The surface soil pH value of the Yangai tea farm was different before and after spring tea picking but was slight. The surface soil pH value of the tea garden before and after spring tea picking ranged from 3.93 to 5.85 (average value of 4.49) and 4.07 to 5.88 (average value of 4.51), respectively (Figs. 2A and 2B). The overall soil pH value different by only 0.02, almost unchanged, indicating spring tea picking had no significant effect on the surface soil pH in the study area.

### Soil exchangeable acid distribution characteristics of surface soil in different periods

The surface soil exchangeable acid in the tea garden was mainly Al^3+^. The surface soil exchangeable H^+^ content before and after spring tea picking ranged from 0.03 to 0.62 (average value 0.27) and 0.04 to 0.61 (average value 0.33) cmol kg^−1^ in the Yangai tea farm, respectively (Figs. 2C and 2D). Their exchangeable Al^3+^ content ranged from 0.09 to 9.32 (average value 6.02) and 0.04 to 8.59 (average value 6.20) cmol kg^−1^, respectively (Figs. 2F and 2E). The content of exchangeable acids (especially Al^3+^) in the soil was too high, which indicated that the exchangeable base in the soil was poor and the soil fertility was low, which was unfavorable for crop health growth. The content distribution of surface soil exchangeable H^+^ and Al^3+^ was not uniform (Figs. S4 and S5).

### SOM distribution characteristics of surface soil in different periods

**Figure 3:**
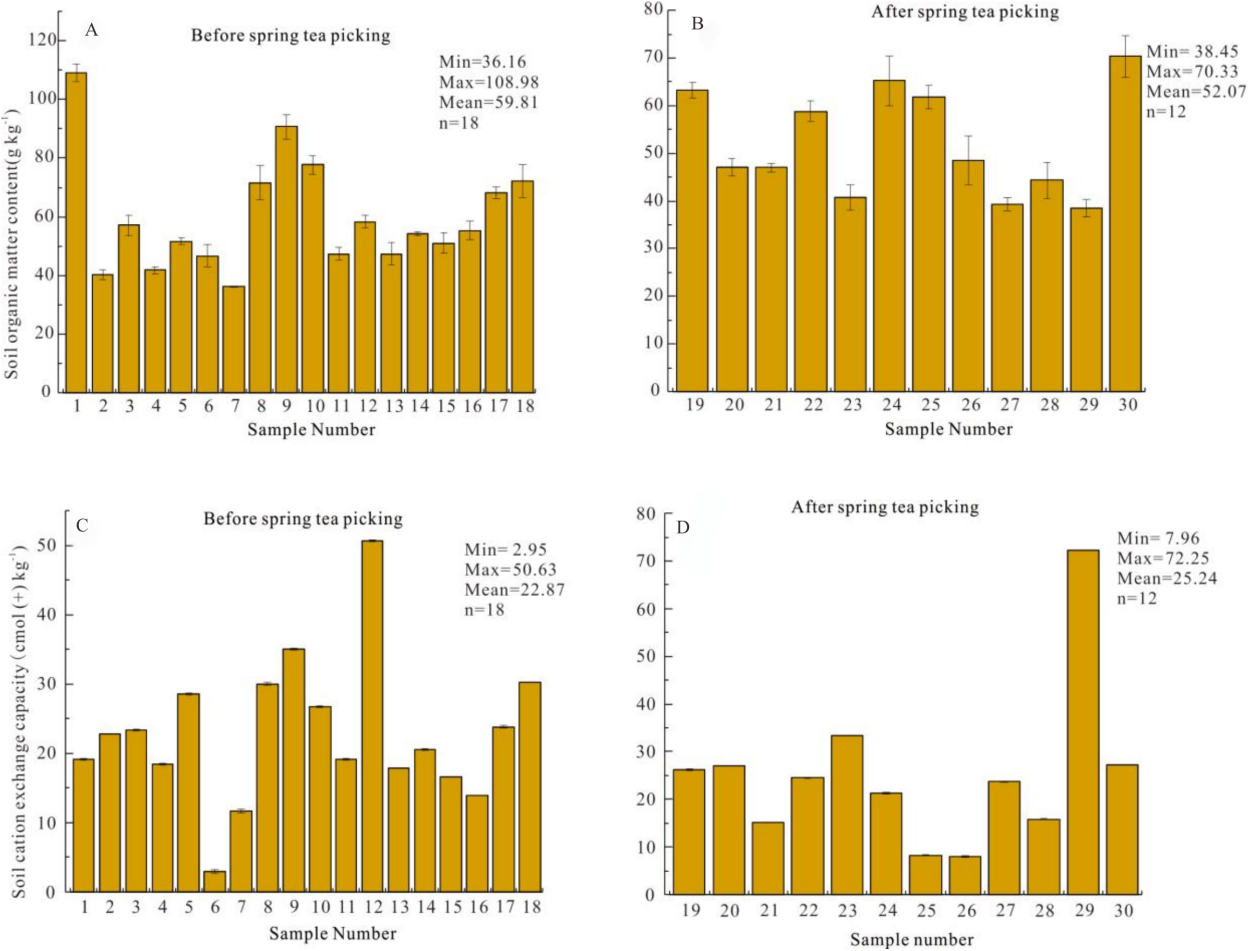
(A) surface SOM contents before spring tea picking; (B) surface SOM contents after spring tea picking; (C) surface soil CEC contents before spring tea picking; (D) surface CEC contents after spring tea picking.

The surface SOM content of the Yangai tea farm was rich, but the content decreased with time. The surface SOM content of the study area was mainly between 50.00 and 70.00 g kg^−1^ (Fig. S6). According to the classification table of SOM content in China’s second soil general survey (Chen et al., 2016), the surface SOM content was the first level as a whole (>40 g kg^−1^), indicating the surface SOM content of the Yangai tea farm was rich. Additionally, the surface SOM content before and after spring tea picking in the Yangai tea farm ranged from 36.16 to 108.98 (average value 59.81) and 38.45 to 70.33 (average value 52.07) g kg^−1^, respectively (Figs. 3A and 3B). This indicated that the surface SOM content before spring tea picking was higher than after spring tea picking in the Yangai tea farm.

### Soil CEC distribution characteristics of surface soil in different periods

**Figure 4:**
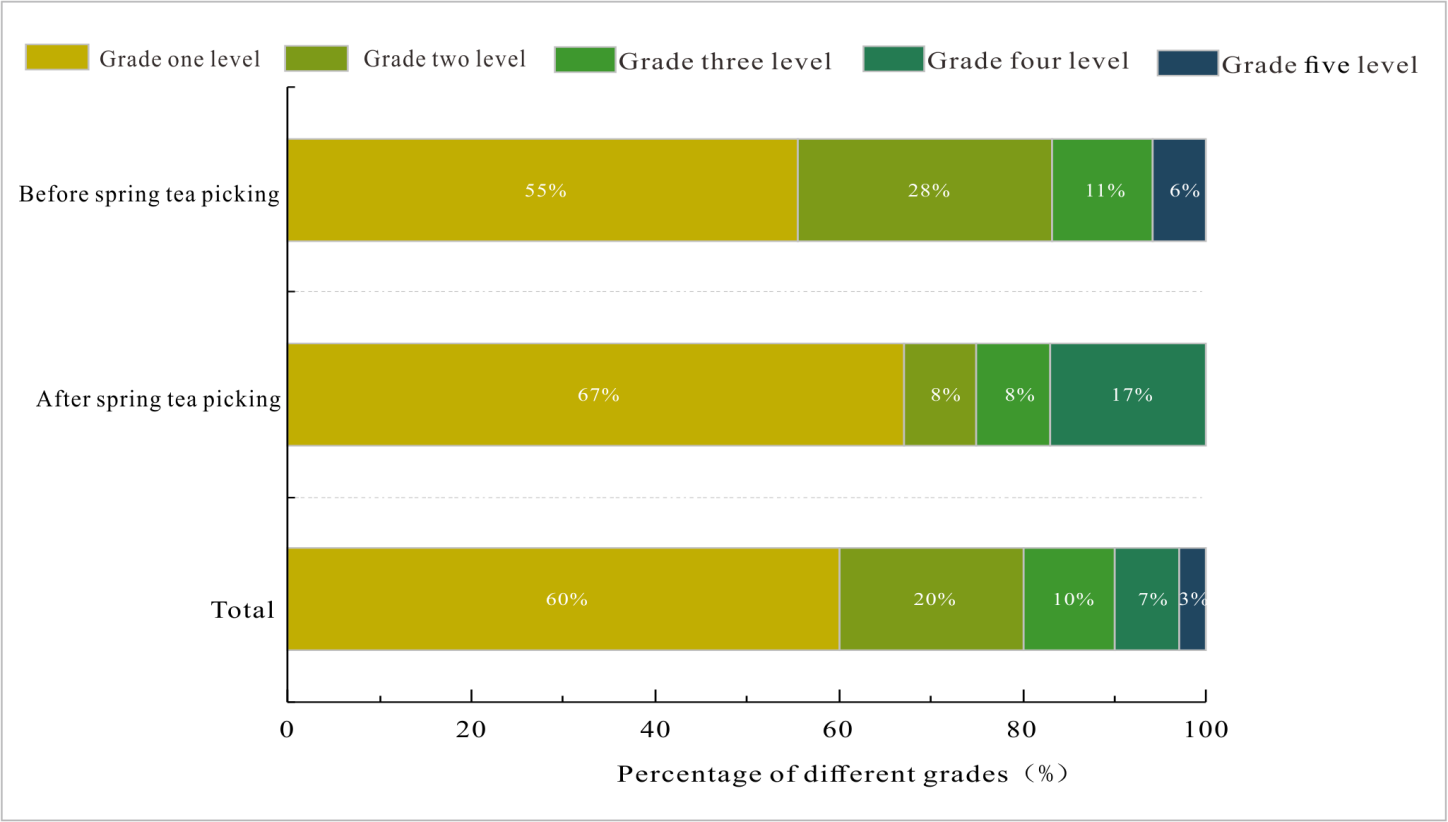
Surface soil CEC grade ratio of the different periods in the Yangai tea farm.

The distribution of surface soil CEC was uneven in the Yangai tea farm, and the content was mainly between 20 and 30 cmol(+)kg^−1^ (Fig. S7). According to the second national soil survey and related standards (Kang et al., 2021), soil CEC was divided into five levels: >20, 15.4∼20, 10.5∼15.4, 6.2∼10.5, and <6.2 cmol(+)kg^−1^ correspond to soil fertility grades one (very strong fertilizer retention), two (strong fertilizer retention), three (medium fertilizer retention), four (weak fertilizer retention), and five (very weak fertilizer retention), respectively. The average surface soil CEC before and after spring tea picking ranged from 2.95 to 50.63 (average 22.87) and 7.96 to 72.25 (average 25.24) cmol(+)kg^−1^ in the Yangai tea farm, respectively (Figs. 3C and 3D). The surface soil CEC in the research area belonged to the grade one level as a whole. These indicated the surface soil fertilizer retention and supply capacity were strong in the Yangai tea farm. Their average soil CEC after spring tea picking was higher than that of before spring tea picking in the research area. In addition, the proportion of soil fertility grade two level and three level before spring tea picking in the Yangai tea farm was 20% and 3% higher than that after spring tea picking, respectively (Fig. 4).

### Soil pH distribution characteristics of soil profiles in different land use types

**Figure 5:**
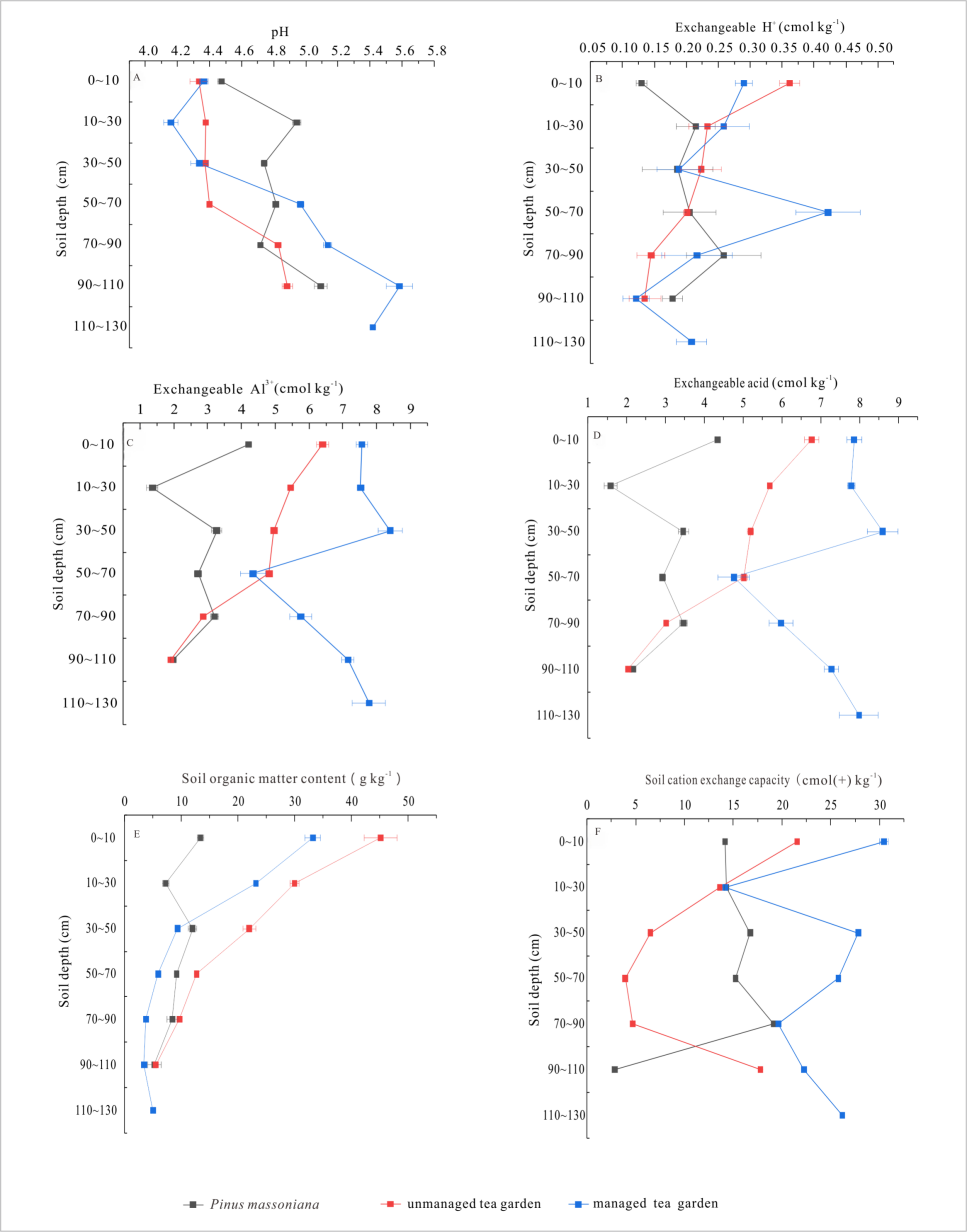
(A) profile soil pH values in different land use types; (B) profile soil exchangeable H^+^ contents in different land use types; (C) profile soil exchangeable Al^3+^ contents in different land use types; (D) profile soil exchangeable acid contents in different land use types; (E) profile SOM contents in different land use types; (F) profile soil CEC contents in different land use types.

Soil pH values of different land use profiles showed an increasing trend with the increase of soil depth in the Yangai tea farm. As shown in Fig. 5A, the profile soil pH value of the managed tea garden at a soil depth of 0 to 30 cm showed a downward trend in the Yangai tea farm, and its change range was more than that of the *Pinus massoniana* forest and the unmanaged tea garden. Soil pH value of the three land use types showed an increasing trend with the increase of soil depth, indicating the tea plant and *Pinus massoniana* forest may lead to soil acidification. Additionally, compared with the soil pH value difference from the surface soil layer to the bottom soil layer of the different land use types, the order was managed tea garden (increasing 1.05) > *Pinus massoniana* forest (increasing 0.62) > unmanaged tea garden (increasing 0.55) (Fig. 5A). These indicated the tea plant and *Pinus massoniana* growth might increase their growth soil acidification, especially in the tea plant of the managed tea garden.

### Soil exchangeable acid distribution characteristics of soil profiles in different land use types

The soil exchangeable acid contents in soil profiles of different land use types in the Yangai tea farm were mainly dominated by exchangeable Al^3+^. Soil exchangeable Al^3+^ contents of soil sections in various land use types were higher than that of exchangeable H^+^ (Figs. 5B and 5C), indicating the soil acidic ion was mainly exchangeable Al^3+^ in the study area. The soil exchangeable Al^3+^ content order in the soil profile was managed tea garden > unmanaged tea garden > *Pinus massoniana* forest (Figs. 5B to 5D).

### SOM distribution characteristics of soil profiles in different land use types

The SOM contents of soil profiles in different land use types decreased with increasing soil depth in the Yangai tea farm. As described in Fig. 5E, the SOM contents of *Pinus massoniana* forest, unmanaged tea garden, and managed tea garden ranged from 5.26 to 13.38 (average value 9.26), 5.50 to 45.15 (20.83), and 3.45 to 33.20 (11.98) g kg^−1^, respectively. Their average SOM content order was unmanaged tea garden > managed tea garden > *Pinus massoniana* forest. We found that the managed tea garden in the study area had mainly organic fertilizer in recent years. These results indicated that SOM content in topsoil in the Yangai tea farm was generally high, which might be affected by plant litter and artificial fertilization. The SOM content in the bottom layer (90–110 cm) of the three land use types was similar (Fig. 5E). Compared with the SOM content difference from the top soil layer to the bottom soil layer of the different land use types, their order was unmanaged tea garden (decreasing 39.64) > managed tea garden (decreasing 28.18) > *Pinus massoniana* forest (decreasing 8.11) (Fig. 5E).

### Soil CEC distribution characteristics of soil profiles in different land use types

Soil CEC in the soil profile of the managed tea garden in the Yangai tea farm was higher. As presented in Fig. 5F, soil CEC in the soil section of the *Pinus massoniana* forest, unmanaged tea garden, and managed tea garden in the study area ranged from 2.86 to 19.11 (average value of 13.73), 3.93 to 21.54 (11.34), and 14.18 to 30.44 (23.75) cmol(+)kg^−1^, respectively. Soil CEC in the soil section of the *Pinus massoniana* forest and unmanaged tea garden showed a fluctuation trend with the increased soil depth. Soil CEC in the soil profile of the managed tea garden decreased drastically during soil depth from 0 to 30 cm, and then the trend of increase-decrease-increase.

### Correlation of soil physicochemical properties

**Figure 6:**
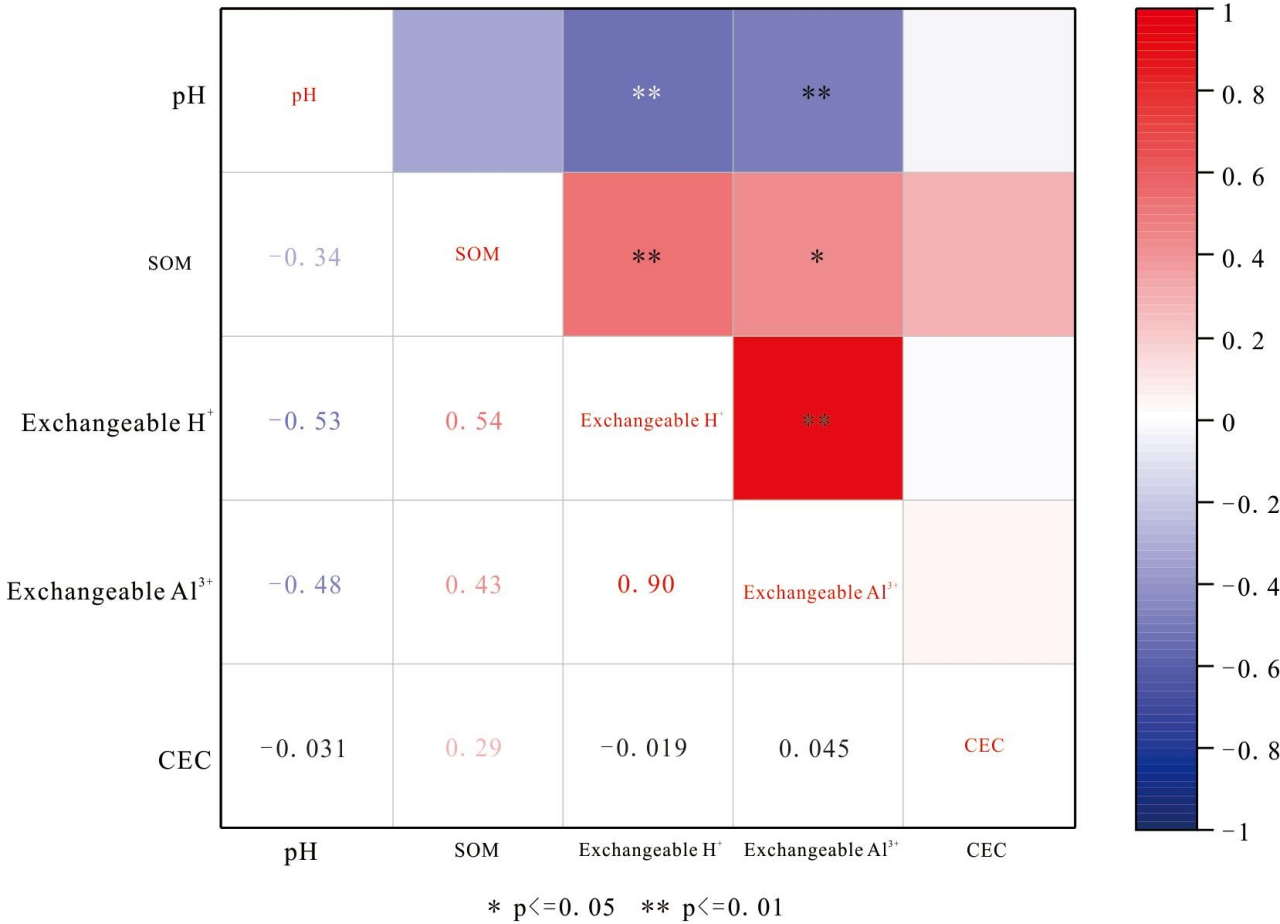
Correlation of surface soil physicochemical properties in the Yangai tea farm.

**Figure 7:**
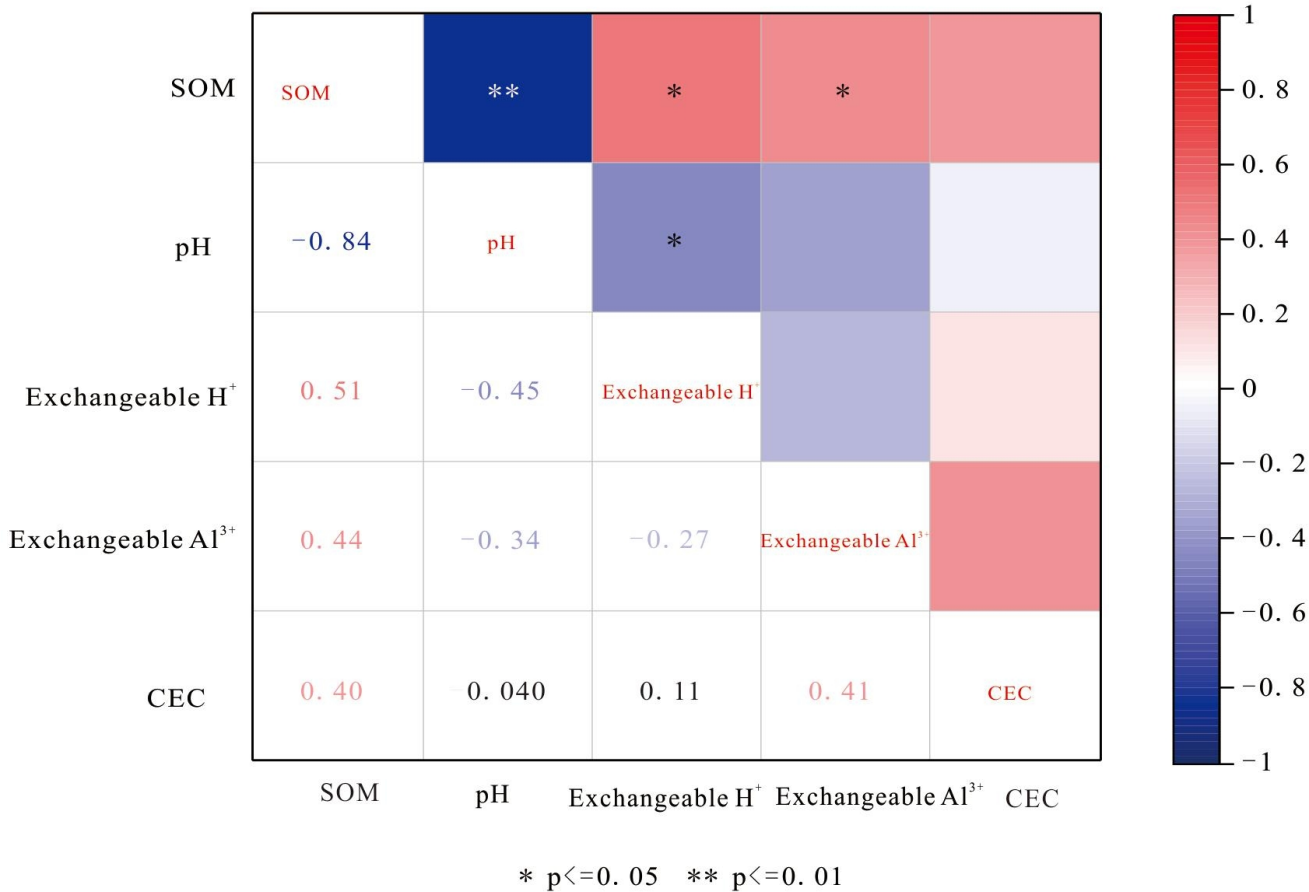
Soil physicochemical property correlation of soil profiles in the Yangai tea farm.

Soil exchangeable H^+^ in the Yangai tea farm showed significant correlations with SOM, soil pH, and soil exchangeable Al^3+^. Soil CEC in the study area showed no significant correlations with other soil properties. As shown in Fig. 6, surface soil exchangeable H^+^ had moderate positive and negative correlations (0.4 ≤|*r*| < 0.6) respective with SOM and soil pH at the 0.01 level and an outstandingly strong positive correlation (0.8 ≤|*r*| < 1) with soil exchangeable Al^3+^ at the 0.01 level. Soil pH also had a moderate negative correlation with soil exchangeable Al^3+^ at the 0.01 level. In contrast, surface soil CEC in the study area showed no significant correlations with other soil properties, and the correlation between soil pH and SOM was not remarkable. As described in Fig. 7, SOM of the soil profile in the Yangai tea farm had an unusually significant negative correlation with soil pH (correlation coefficient: −0.84, *P* < 0.01) and had remarkably notable positive correlations with soil exchangeable H^+^ and soil exchangeable Al^3+^ respective with correlation coefficients of 0.51 (*P* <0.01) and 0.44 (*P* <0.01). Soil pH had a significant negative correlation with exchangeable H^+^ (correlation coefficient: −0.45, *P* < 0.05). Additionally, soil CEC of the soil profile showed no significant correlations with other physicochemical properties.

## Discussion

### Soil pH spatial distribution characteristics

The overall average soil pH value before and after spring tea picking in the Yangai tea farm was lower than the national average soil pH value (4.68) (Yan et al., 2020), indicating that the soil acidification degree of the Yangai tea farm was higher than that of the national tea garden. The surface soil pH values in the Yangai tea farm were mainly between 4.00 and 4.50, but the surface soil with a pH value in the middle area was between 4.5 and 5.5 (Fig. S3). Surface soil pH values in the range of <4.5, 4.5−5.5, and >5.5 before spring tea picking in the research area accounted for 61.1%, 33.3%, and 5.6%, respectively. Surface soil pH values in the range of <4.5, 4.5−5.5, and >5.5 after spring tea picking in the research area accounted for 81.8%, 9.1%, and 9.1%, respectively. These were consistent with the previous studies that found most soil pH values in Guizhou Province were < 4.5 (Yan et al., 2020). At the same time, the proportion of the optimum soil pH value range (4.5–5.5) (Ye et al., 2025) suitable for the growth of tea plants decreased significantly (about 24.2%), indicating that the change of surface soil pH value did not suit for the tea plant growth in the study area. Tea planting might lead to soil acidification (Ye et al., 2025). However, the surface soil pH value had little change before and after spring tea picking in the Yangai tea farm, which might be affected by using organic fertilizer (Yan et al., 2018; Zeng et al., 2017). In addition, the proportion of soil suitable for the tea plant growth (4.5 < soil pH value < 5.5) showed a decreasing trend, indicating the Yangai tea farm should be managed accurately by different areas with varying soil pH levels in the later stage.

Tea plant and *Pinus massoniana* tree growth might lead to soil acidification of their growth soil, especially in the tea plant of the managed tea garden. The soil pH value difference from the surface soil layer to the bottom soil layer of the managed tea garden, *Pinus massoniana* forest, and unmanaged tea garden in the study area increased by 1.05, 0.62, and 0.55, respectively (Fig. 5A). It indicated that the soil acidification in the upper part of the soil profile in the managed tea garden was more serious than in the *Pinus massoniana* forest and unmanaged tea garden. The soil pH value of the soil profile changing trend of the managed tea garden in this study was similar to the previous research (Alekseeva et al., 2010). Soil acidification occurred within 70 cm of the tea garden in Nanjing, China, and the soil pH value at 0–17 cm was relatively low (Alekseeva et al., 2010). It might be due to loosening the surface soil, increasing soil aeration, accelerating soil organic matter decomposition, increasing the activity of tea plant roots, enhancing aluminum aggregation ability, ultimately leading to more severe surface soil acidification in tea gardens through tea garden management measures (*i.e.,* fertilization, weeding, and cultivation).

### Soil exchangeable acid spatial distribution characteristics

Soil exchangeable acid in the Yangai tea farm was mainly dominated by Al^3+^. Before and after spring tea picking, the surface soil exchangeable H^+^ content ranged from 0.03 to 0.62 (average value 0.27) and 0.04 to 0.61 (average value 0.33) cmol kg^−1^ in the Yangai tea farm, respectively (Figs. 2C and 2D). Their exchangeable Al^3+^ content ranged from 0.09 to 9.32 (average value 6.02) and 0.04 to 8.59 (average value 6.20) cmol kg^−1^, respectively (Figs. 2F and 2E). Soil exchangeable Al^3+^ contents of soil sections in various land use types were higher than that of exchangeable H^+^ (Figs. 5B and 5C), indicating the soil acidic ion was mainly exchangeable Al^3+^ in the study area. The concentration distribution of the surface soil exchange Al^3+^ and H^+^in the Yangai tea farm was similar, decreasing first and then increasing along the southeast to northwest direction (Figs. S4 and S5). In addition, the soil exchangeable Al^3+^ content order of the soil profile in the Yangai tea farm was managed tea garden >unmanaged tea garden >*Pinus massoniana* forest (Fig. 5C). The managed tea garden was more likely to increase the soil exchangeable Al^3+^ content than the *Pinus massoniana* forest. The exchangeable Al^3+^ caused to soil acidification (Yan et al., 2020). Therefore, the main reason that the soil acidification rate of the managed tea garden was higher than that of the *Pinus massoniana* forest is tea plant growth could activate aluminum in the soil and accelerate the soil acidification rate.

### SOM spatial distribution characteristics

The surface SOM content before spring tea picking in the Yangai tea farm was higher than that after. The surface SOM content before and after spring tea picking in the Yangai tea farm ranged from 36.16 to 108.98 (average value 59.81) and 38.45 to 70.33 (52.07) g kg^−1^, respectively (Figs. 3A and 3B). The concentration distribution of the surface SOM in the Yangai tea farm showed a gradually increasing trend from southeast to northwest (Fig. S6). The soil microorganisms could promote the degradation of SOM through catabolism (Liang, Schimel & Jastrow, 2017). The flood season in mountainous areas of Guizhou Province was from April to September (Zhao, 2020). The average winter temperature and spring temperature in Guiyang City were generally 5.0 °C–12.0 °C and 8.0 °C–16.0 °C, respectively. Winter and early spring might limit microbial metabolism and SOM decomposition due to their relatively low temperature. However, as temperatures rose in late spring, microbial activity likely increased, it increased SOM decomposition. These might lead to the SOM content after spring tea picking in the Yangai tea farm was lower than that before spring tea picking. Therefore, the organic matter in the soil of the Yangai tea farm would decompose and be consumed more during the spring, summer, and autumn tea-picking periods than in winter. It should be supplemented with moderate organic fertilizers after tea-picking in the research area.

With the increase of soil depth, the SOM content of different land use types decreases, and the change range of soil profile in the unmanaged and managed tea garden was higher than that in the *Pinus massoniana* forest soil (Fig. 5E). Sugars, amino acids, and phenolic compounds secreted by plant roots were the primary sources of SOM (Zhou, Hou & Mofan, 2023). Some studies have shown that SOM content decreases with the increase in soil depth (Li et al., 2015; Wang et al., 2021; Zhu et al., 2017). Therefore, the difference between the root exudates of the tea plant and *Pinus massoniana* might be why the change range of soil profile in the *Pinus massoniana* forest soil profile was smaller than that in the tea garden profile, and the specific exudates need further investigation.

The interaction and mutual influence were among soil pH value, microbial activity, and SOM. As soil pH decreased, microbial communities shifted towards acid-tolerant species, which might have lower efficiency in decomposing SOM, leading to slower organic matter turnover. Additionally, increased soil acidification often correlated with enhanced aluminum toxicity, which inhibited microbial activity and root growth, further restricting SOM accumulation and nutrient cycling (Alekseeva et al., 2010; Zhang et al., 2024). Soil microbial activity was also strongly influenced by soil acidification levels. Soil CO_2_ evolution was significantly lower in more acidic plots (pH < 4.5) compared to less acidic areas, indicating a decline in microbial respiration under highly acidic conditions. Dehydrogenase activity showed a similar trend, with enzyme activity being highest in moderately acidic soils (pH 5.0–5.5) and declining in severely acidified soils (pH < 4.0) (Tang et al., 2024). These results aligned with the previous studies: soil microbial metabolic activity was suppressed under extreme soil acidification, reducing SOM decomposition and nutrient cycling (Feketeová, Hrabovský & Šimkovic, 2021). Therefore, the increased soil acidification in tea plantations exacerbated SOM decomposition through biological and chemical pathways, necessitating careful soil management to maintain fertility and sustainability.

### Soil CEC spatial distribution characteristics

Fertilization might be the reason for increasing the soil CEC in the study area. The distribution of surface soil CEC was uneven in the Yangai tea farm, and the content was mainly between 20 and 30 cmol(+)kg^−1^ (Fig. S7). The surface soil CEC of vegetable gardens requiring perennial tillage and fertilization was higher than that of paddy land, woodland, and dryland (Bi et al., 2023), and its CEC was similar to the surface soil CEC of the Yangai tea farm (about > 20 cmol(+)kg^−1^). The average surface soil CEC before and after spring tea picking ranged from 2.95 to 50.63 (average 22.87) and 7.96 to 72.25 (average 25.24) cmol(+)kg^−1^ in the Yangai tea farm, respectively (Figs. 3C and 3D). The concentration distribution of the surface soil CEC in the Yangai tea farm showed a gradually increasing trend from southwest to northeast (Fig. S7). The proportion of soil CEC with grade one level fertility preservation before and after spring tea picking was 55% and 67%, respectively, about 12% higher. The surface soil CEC after spring tea picking was generally higher than before in the Yangai tea farm, which might be caused by using organic fertilizer after spring tea picking (Domingues et al., 2020), the application of SOM before spring tea picking quickly composed during the spring tea picking period, leading to increase soil mineral nutrients, humus, and CEC. Soil CEC was also affected by the topography and landform (Han, Jang & Chung, 2017). The Yangai tea farm, with the humid and hot climate of subtropical soil, resulted in very strong leaching of cations (Yu et al., 2020). Additionally, the surface soil CEC of the managed tea garden was higher than that of the unmanaged tea garden and *Pinus massoniana* forest, which might be affected by fertilization. The soil CEC trend of soil profile at soil depth from 0 to 50 cm in the managed tea garden was consistent with previous research (Bi et al., 2023). Bio-organic fertilizers might increase soil CEC (Ansari & Mahmood, 2017; Lu et al., 2019). It might be affected by long-term cultivation and fertilization. Therefore, in the later stage of tea garden management at the Yangai tea farm, there should be attention to the timely supply of fertilizers.

### Correlation of soil physicochemical properties

**Figure 8:**
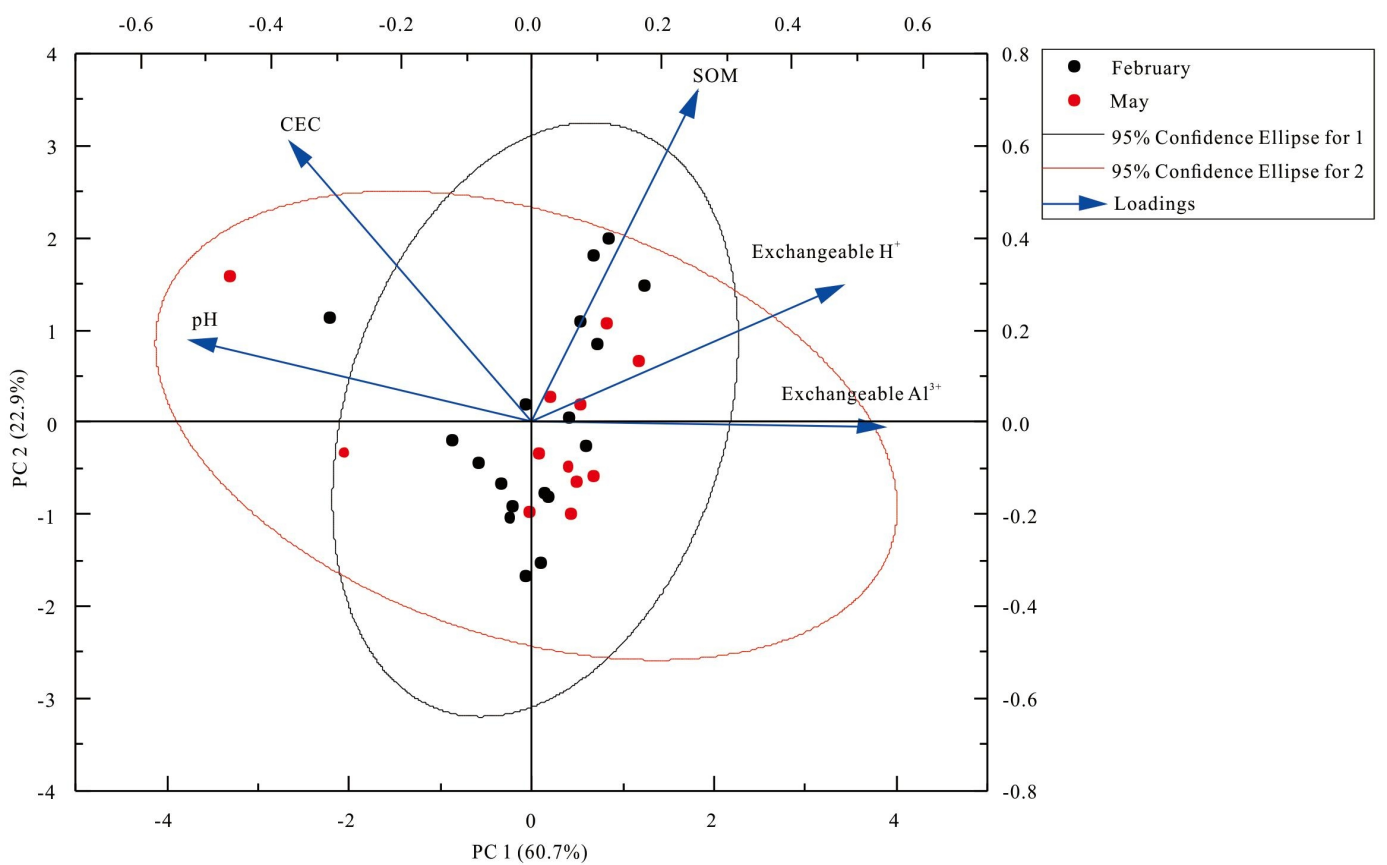
Principal component analysis (PCA) of surface soil physicochemical properties in the Yangai tea farm.

Surface soil pH, exchangeable Al^3+^, and exchangeable H^+^ in the Yangai tea farm were negatively correlation (*P* < 0.01) (Fig. 6). It indicated the surface soil exchangeable Al^3+^ and exchangeable H^+^ content was higher, its soil pH was lower, and the soil acidification of the tea garden was more serious. However, the correlation is different between surface soil pH and exchangeable Al^3+^ in the same place with time (Gillespie, Antonangelo & Zhang, 2021; Lollato et al., 2019). Soil exchangeable Al^3+^ changes were different trends when soil pH was different places changes (Gillespie, Antonangelo & Zhang, 2021). The correlation of soil physicochemical properties between soil profile and surface soil was different (Figs. 6 and 7). Human management (*i.e.,* fertilization (Wu & Chen, 2023) and pesticide application (Lan et al., 2019) would affect the soil physicochemical properties, which might be the reason for the physicochemical properties correlation of surface soil being different from that of the soil profile. In addition, PC1 and PC2 had variance contribution rates of 60.74% and 22.87% in the Yangai tea farm, respectively (Table S2), with a cumulative contribution rate of 83.62%. It indicated that these two principal components could effectively represent the variation information in the original data. Loading analysis revealed a significant positive correlation between SOM and PC2 (loading of 0.72) in the study area, while its correlation with PC1 was weak (loading of 0.26). Soil pH exhibited a negative correlation with PC1 (loading of −0.52) and a weak positive correlation with PC2 (loading of 0.18). Both soil exchangeable H^+^ and exchangeable Al^3+^ were positively correlated with PC1 respective loadings of 0.48 and 0.54 but showed weak correlations with PC2. Soil CEC had a positive correlation with PC2 (loading of 0.61) and a negative correlation with PC1 (loading of −0.37). These results suggested that PC1 mainly reflected the variations in soil exchangeable H^+^, exchangeable Al^3+^, and pH, while PC2 was primarily associated with SOM and CEC. Therefore, temporal variations and inherent differences between soil environments might influence the correlation between soil pH and exchangeable Al^3+^ (Table S3 and Fig. 8).

## Conclusions

In the Yangai tea farm, there was severe surface soil acidification in the tea garden, but had a strong fertilizer retention capacity. The soil pH value, exchangeable Al^3+^ content, and exchangeable H^+^ content ranged from 3.95 to 5.88 (average value 4.50), 0.04 to 9.32 (6.11) cmol kg^−1^, and 0.03 to 0.62 (0.30) cmol kg^−1^, respectively. The SOM content ranged from 36.16 to 108.98 (average value 55.94) g kg^−1,^ and the soil CEC varied between 2.95 to 72.25 (average value 24.06) cmol(+)kg^−1^, respectively. Surface soil exchangeable acid and CEC contents were higher after spring tea picking than those before spring tea picking in the Yangai tea farm, while their SOM content was just the opposite. Therefore, it was recommended to supplement organic fertilizers after tea-picking to replenish lost nutrients and improve soil structure because surface SOM of the Yangai tea farm would decompose and be consumed more during the spring, summer, and autumn tea-picking periods than in winter.

The fertilizer holding capacity of the managed tea plantation soil was better than that of the *Pinus massoniana* forest soil and the unmanaged tea plantation soil. The average value order of the soil CEC in different land use profiles was the managed tea garden (23.75 cmol(+)kg^−1^) > *Pinus massoniana* forest (13.73 cmol(+)kg^−1^) > unmanaged tea garden (11.34 cmol(+)kg^−1^). With the increase of soil depth, the SOM content of different land use types decreases, and the change range of soil profile in the unmanaged and managed tea garden is higher than that in the *Pinus massoniana* forest soil. These indicated that intensive management practices improved nutrient retention.

Tea plant and *Pinus massoniana* growth might lead to their growth soil acidification, especially in the tea plant of the managed tea garden. The soil pH value from the surface soil layer to the bottom soil layer of the managed tea garden, *Pinus massoniana* forest, and unmanaged tea garden in the Yangai tea farm increased by 1.05, 0.62, and 0.55, respectively (Fig. 5A). The soil exchangeable Al^3+^ content order of soil profile in three land use types in the study area was the managed tea garden > unmanaged tea garden > *Pinus massoniana* forest. These indicated that the soil acidification in the upper part of the soil profile in the managed tea garden was more serious than in the *Pinus massoniana* forest and unmanaged tea garden. It might be due to the soil acidification rate of the managed tea garden being higher than that of the *Pinus massoniana* forest since tea plant growth could activate aluminum in the soil and accelerate the soil acidification rate. Therefore, future tea plantation management should be focused on soil conservation strategies, such as SOM amendments and soil pH regulation, to mitigate excessive soil acidification and sustain soil productivity. For example, liming biochar, compost, or manure could enhance soil microbial activity and buffer against further pH decline. Intercropping tea plants with nitrogen-fixing species such as clover or alfalfa could help improve SOM levels while reducing the dependency on synthetic fertilizers. Government-supported soil testing programs and region-specific fertilizer recommendations could further assist tea farmers in adopting more sustainable soil management practices, such as microbial-assisted soil rehabilitation (*i.e.,* beneficial bacterial and fungal inoculants).

Although this study provided valuable insights into soil fertility dynamics of the surface soil in different periods and the soil profile in land use types, some limitations should be acknowledged. Soil microbial properties (*i.e.,* microbial respiration (CO_2_ evolution), enzymatic activity (dehydrogenase and phosphatase), and microbial biomass) also played a crucial role in nutrient cycling and organic matter decomposition in tea plantations (Delgado & Gómez, 2024; Ogwu et al., 2024). This study failed to fully consider these indicators, which left gaps in understanding microbial diversity and functional roles. Spatial interpolation was based on a limited number of sampling points, and increasing the sample density could improve the accuracy of soil property distribution models. Despite these limitations, this study laid a foundation for future research, emphasizing the need for comprehensive soil health assessments integrating biological, chemical, and microbial community analyses.

## Supplemental Information

10.7717/peerj.20341/supp-1 Supplemental Information 1 Soil profile of managed tea garden, unmanaged tea garden and *Pinus massoniana* forest in the Yangai tea farm (A) unmanaged tea garden; (B) the soil profile of unmanaged tea garden; (C) managed tea garden; (D) the soil profile of managed tea garden; (E) *Pinus massoniana*; (F) the soil profile of *Pinus massoniana* forest

10.7717/peerj.20341/supp-2 Supplemental Information 2 Experimental design process of this study

10.7717/peerj.20341/supp-3 Supplemental Information 3 Surface soil pH value distribution of the Yangai tea farm

10.7717/peerj.20341/supp-4 Supplemental Information 4 Surface soil exchange Al^3+^ distribution of the Yangai tea farm

10.7717/peerj.20341/supp-5 Supplemental Information 5 Surface soil exchange H^+^ distribution of the Yangai tea farm

10.7717/peerj.20341/supp-6 Supplemental Information 6 Surface SOM distribution of the Yangai tea farm

10.7717/peerj.20341/supp-7 Supplemental Information 7 Surface soil CEC distribution of the Yangai tea farm

10.7717/peerj.20341/supp-8 Supplemental Information 8 Sampling point coordinates in the Yangai tea farm

10.7717/peerj.20341/supp-9 Supplemental Information 9 Principal component analysis (PCA) results of surface soil physicochemical properties in the study area

10.7717/peerj.20341/supp-10 Supplemental Information 10 Factor loading of surface soil physicochemical properties by Principal Component Analysis in the study area

10.7717/peerj.20341/supp-11 Supplemental Information 11 Measured values of four kinds of soil physical and chemical properties

## Additional Information and Declarations

### Data Availability

The following information was supplied regarding data availability: The raw measurements are available in the Supplementary Files.
