# Supplementary figures and images for: Interaction of soil pH, organic matter, exchangeable acidity, and cation exchange capacity in a managed tea farm
Source: PeerJ. 2025 Nov 24;13:e20341. doi: 10.7717/peerj.20341 (PMC12659706; doi:10.7717/peerj.20341)

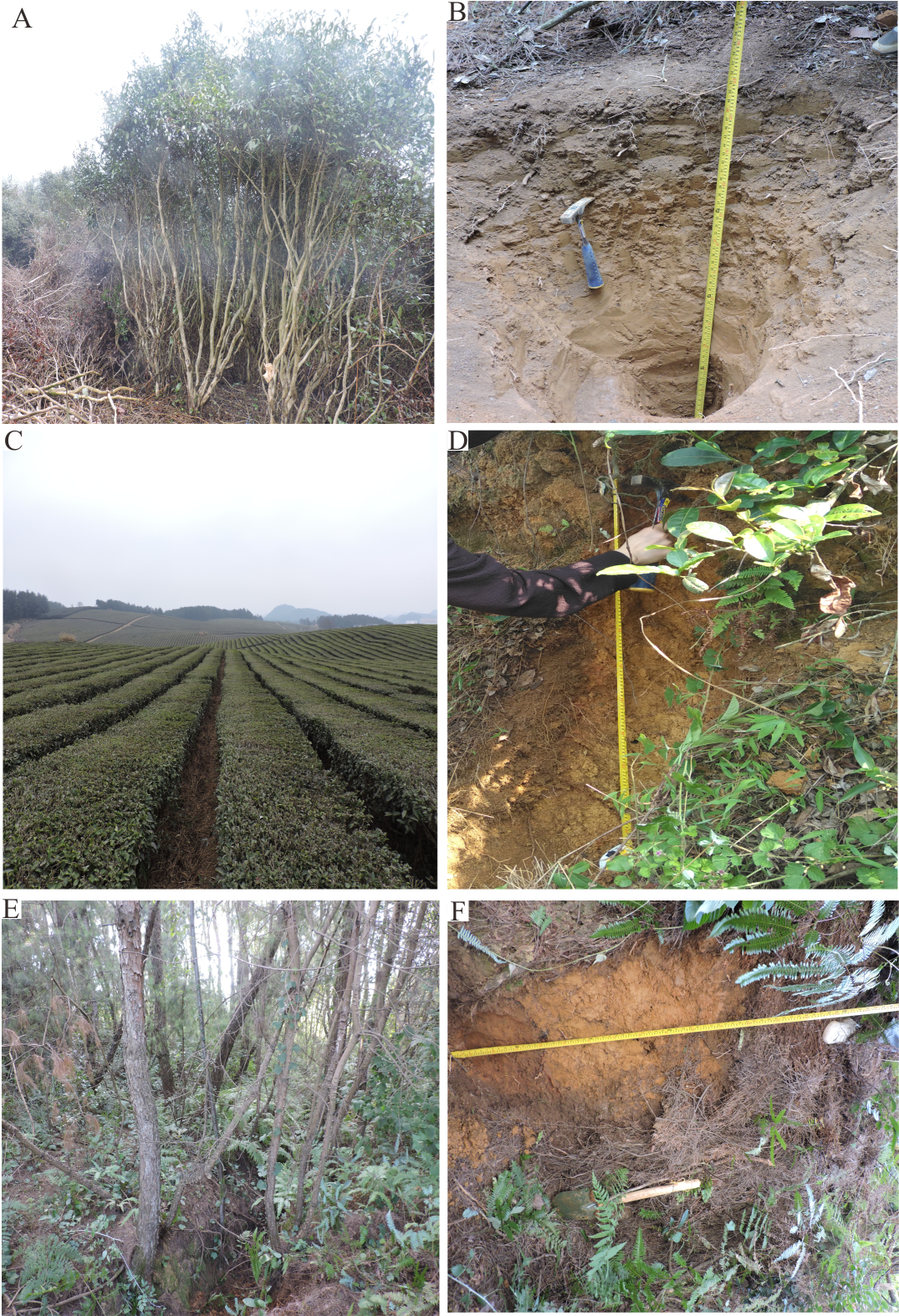

Supplement: Supplemental Information 1 — (A) unmanaged tea garden; (B) the soil profile of unmanaged tea garden; (C) managed tea garden; (D) the soil profile of managed tea garden; (E) Pinus massoniana; (F) the soil profile of Pinus massoniana forest [file peerj-13-20341-s001.png]

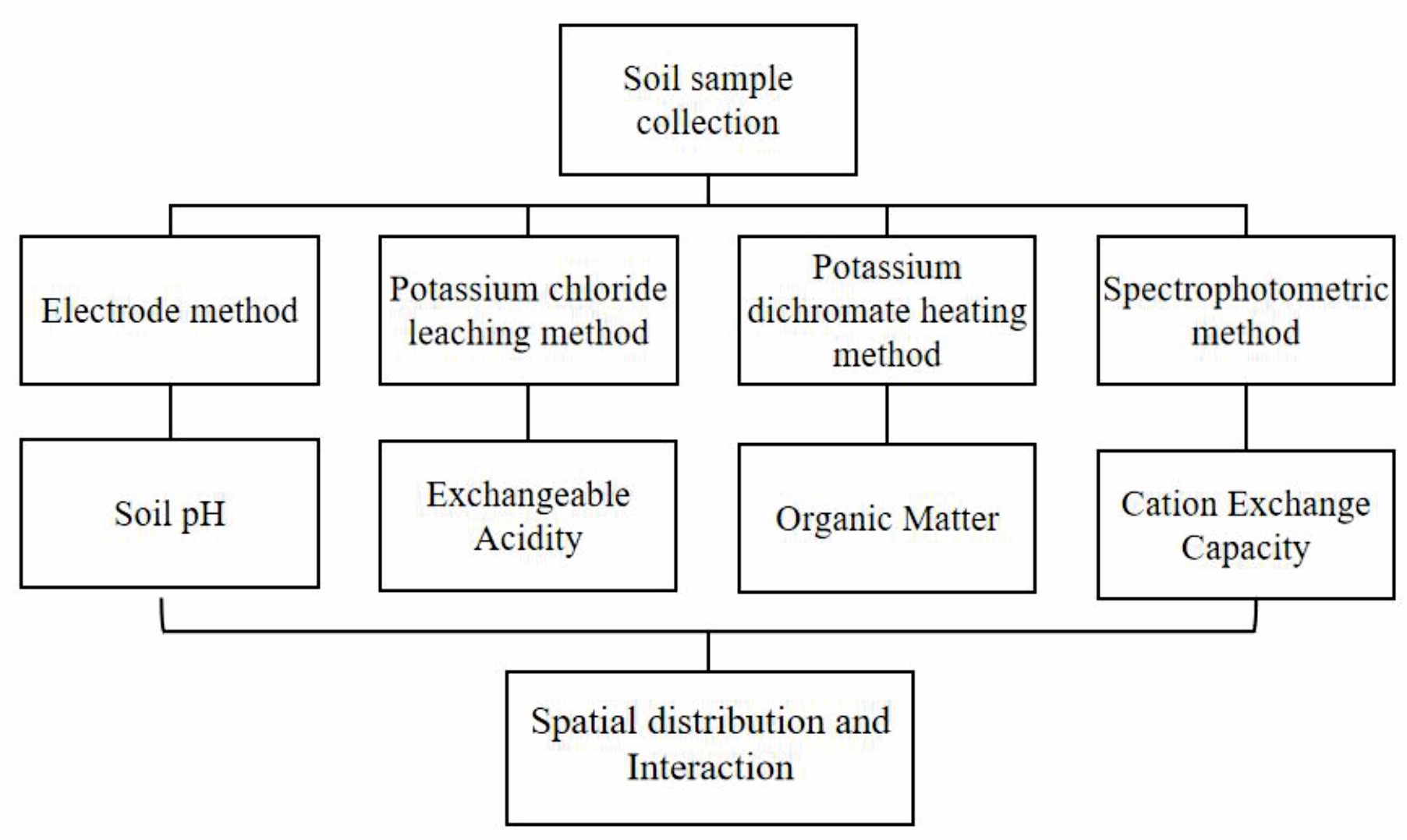

Supplement: Supplemental Information 2 [file peerj-13-20341-s002.jpg]

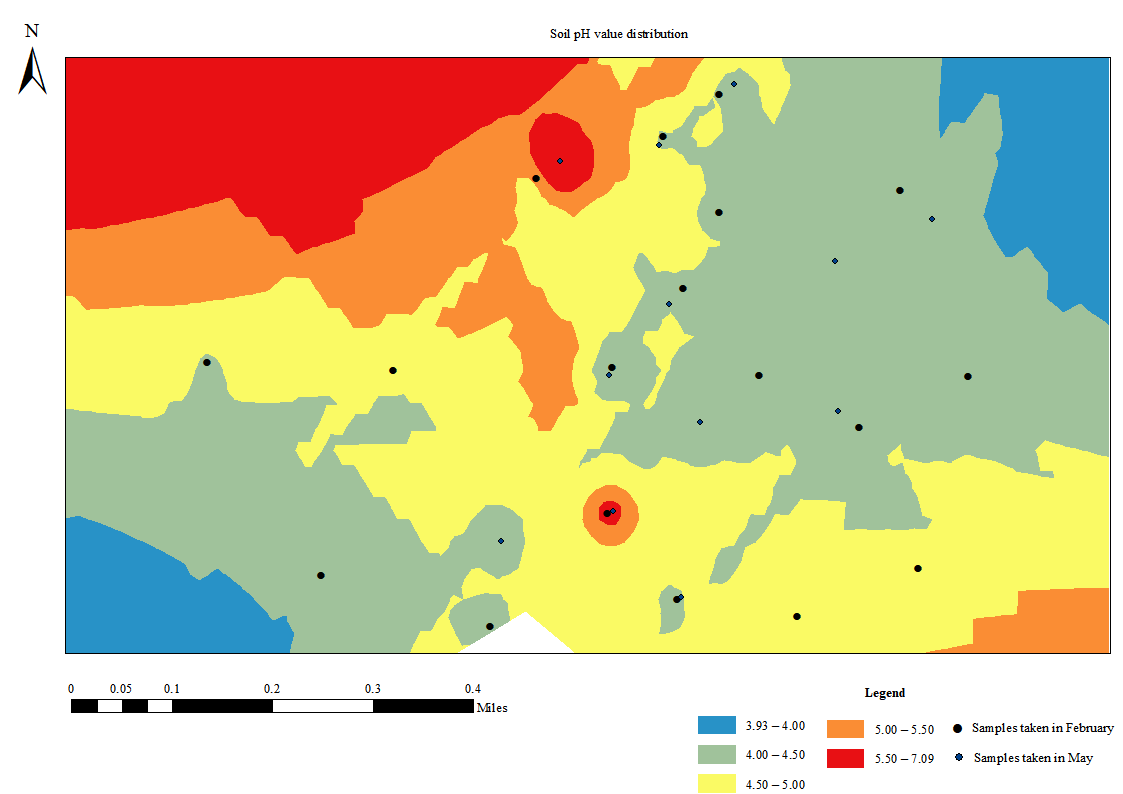

Supplement: Supplemental Information 3 [file peerj-13-20341-s003.png]

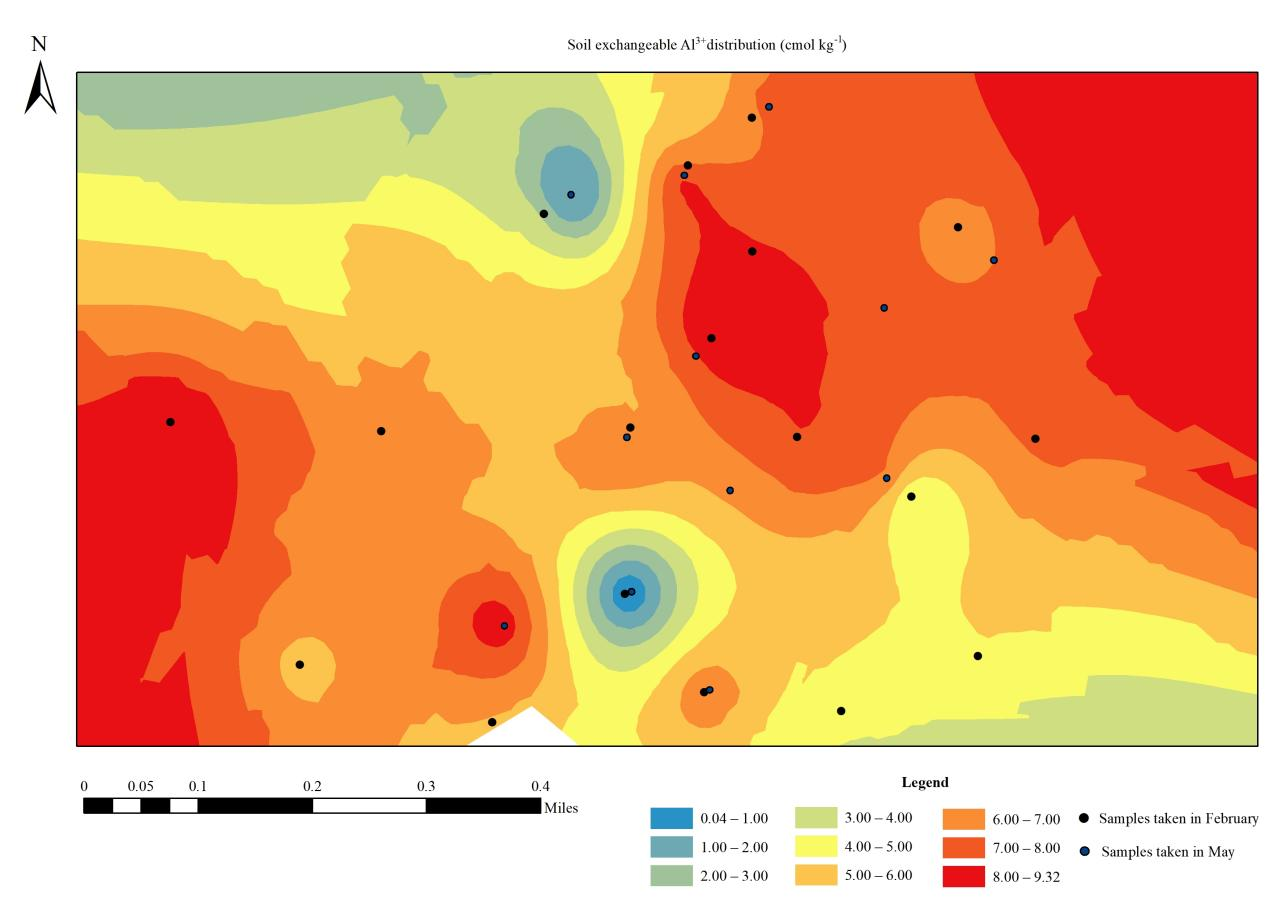

Supplement: Supplemental Information 4 [file peerj-13-20341-s004.png]

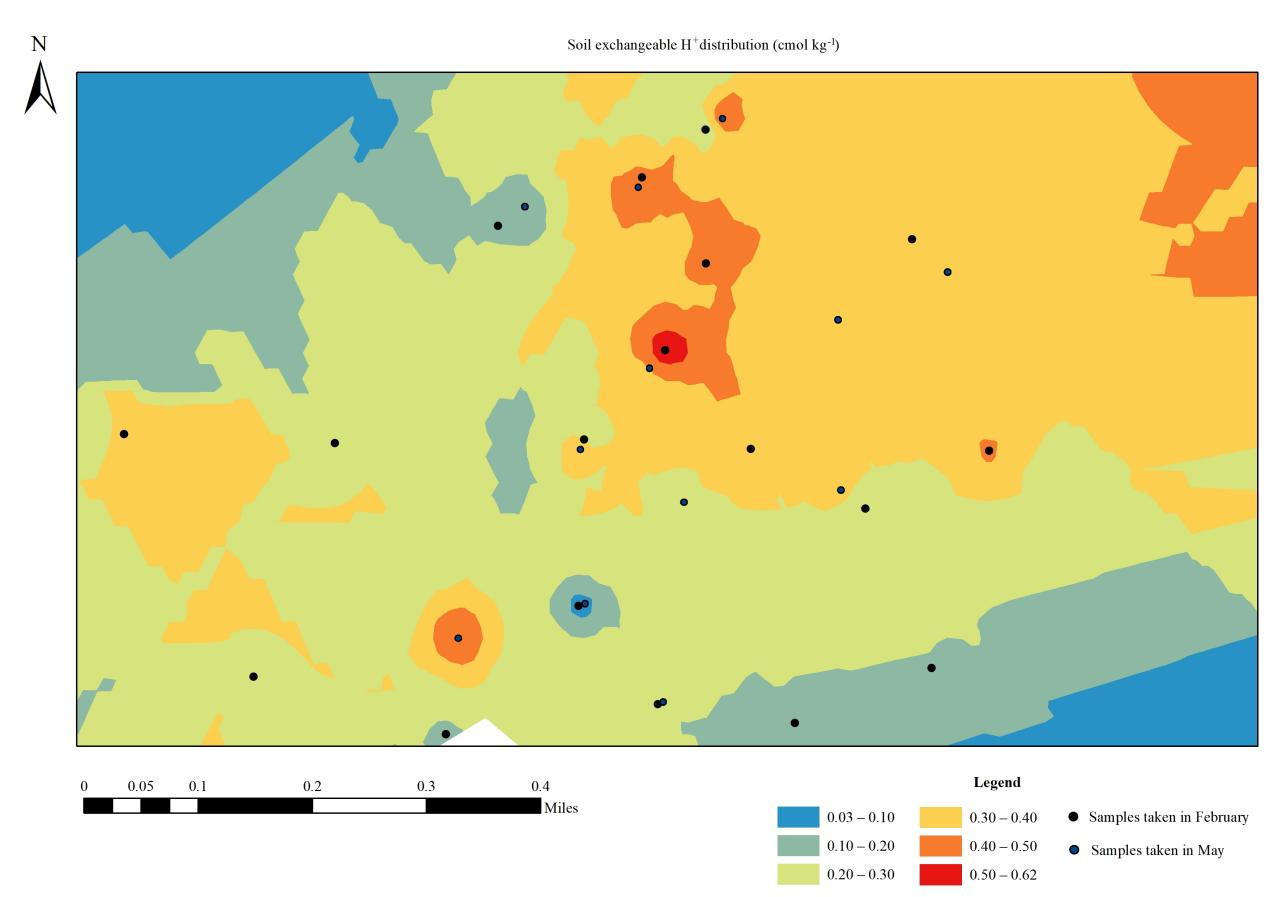

Supplement: Supplemental Information 5 [file peerj-13-20341-s005.png]

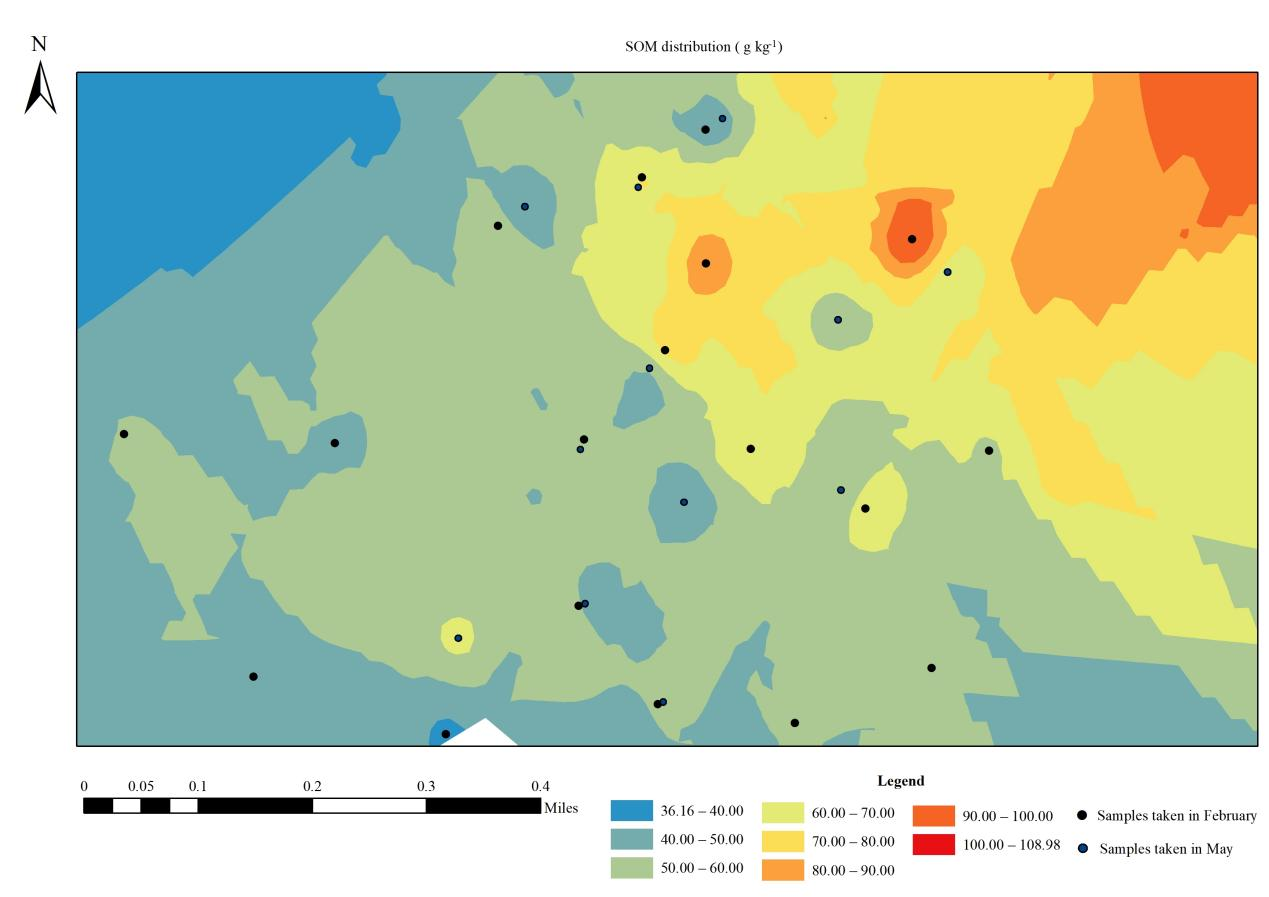

Supplement: Supplemental Information 6 [file peerj-13-20341-s006.png]

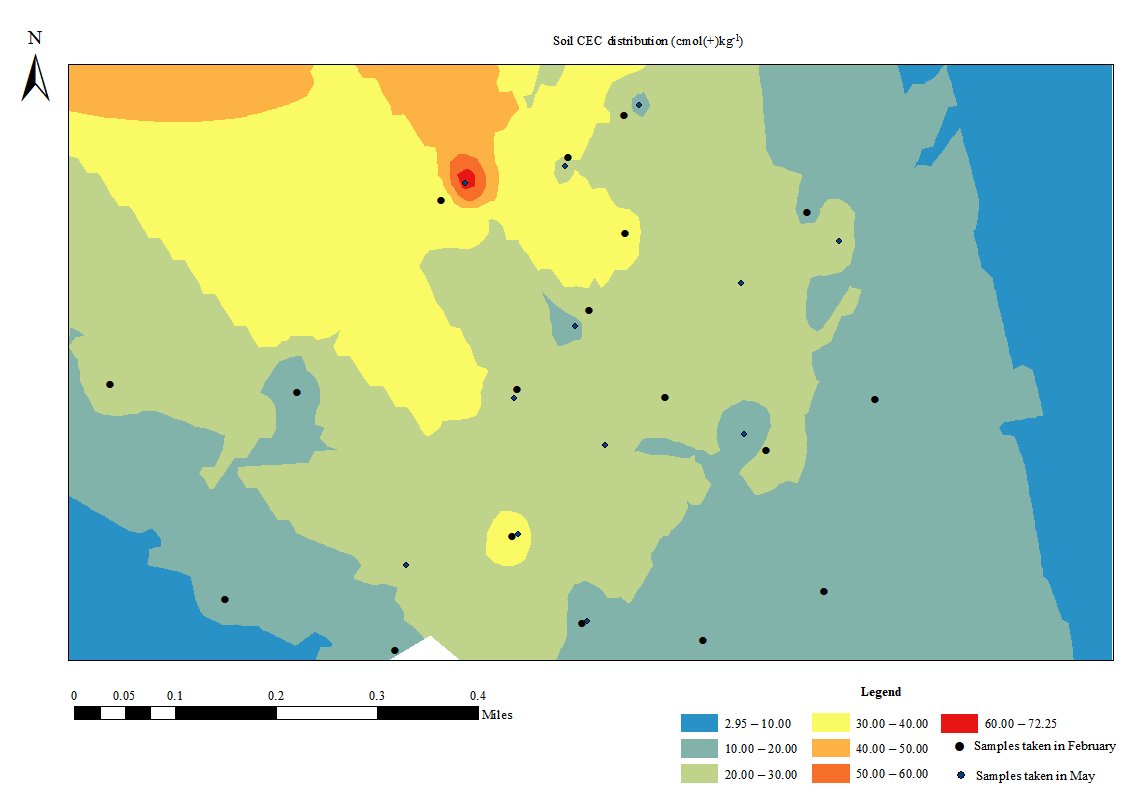

Supplement: Supplemental Information 7 [file peerj-13-20341-s007.png]
