# Supplementary material for: Interaction of soil pH, organic matter, exchangeable acidity, and cation exchange capacity in a managed tea farm
Source: PeerJ. 2025 Nov 24;13:e20341. doi: 10.7717/peerj.20341 (PMC12659706; doi:10.7717/peerj.20341)
Supplement: Supplemental Information 8 [file peerj-13-20341-s008.docx]

Table S1 Sampling point coordinates in the Yangai tea farm

| Sample type | | Sampling number | Northern latitude | East longitude |
| --- | --- | --- | --- | --- |
| Soil profiles | Land use type | *Pinus massoniana* forest | 26.39 | 106.53 |
|  |  | Unmanaged tea garden | 26.39 | 106.53 |
|  |  | Managed tea garden | 26.39 | 106.53 |
| Surface soil | Before spring tea pick | 1 | 26.39 | 106.53 |
|  |  | 2 | 26.39 | 106.53 |
|  |  | 3 | 26.39 | 106.52 |
|  |  | 4 | 26.38 | 106.52 |
|  |  | 5 | 26.39 | 106.52 |
|  |  | 6 | 26.39 | 106.52 |
|  |  | 7 | 26.38 | 106.52 |
|  |  | 8 | 26.39 | 106.53 |
|  |  | 9 | 26.39 | 106.53 |
|  |  | 10 | 26.39 | 106.53 |
|  |  | 11 | 26.39 | 106.53 |
|  |  | 12 | 26.39 | 106.53 |
|  |  | 13 | 26.38 | 106.53 |
|  |  | 14 | 26.38 | 106.53 |
|  |  | 15 | 26.38 | 106.53 |
|  |  | 16 | 26.39 | 106.53 |
|  |  | 17 | 26.39 | 106.53 |
|  |  | 18 | 26.39 | 106.53 |
|  | After spring tea pick | 19 | 26.39 | 106.53 |
|  |  | 20 | 26.39 | 106.53 |
|  |  | 21 | 26.39 | 106.53 |
|  |  | 22 | 26.39 | 106.53 |
|  |  | 23 | 26.39 | 106.53 |
|  |  | 24 | 26.38 | 106.52 |
|  |  | 25 | 26.38 | 106.53 |
|  |  | 26 | 26.39 | 106.53 |
|  |  | 27 | 26.39 | 106.53 |
|  |  | 28 | 26.39 | 106.53 |
|  |  | 29 | 26.39 | 106.52 |
|  |  | 30 | 26.39 | 106.53 |

Note: The sampling point coordinates of soil profiles in different land use types is the midpoint of the collected mixed sample.
