# Supplementary material for: Interaction of soil pH, organic matter, exchangeable acidity, and cation exchange capacity in a managed tea farm
Source: PeerJ. 2025 Nov 24;13:e20341. doi: 10.7717/peerj.20341 (PMC12659706; doi:10.7717/peerj.20341)
Supplement: Supplemental Information 9 [file peerj-13-20341-s009.docx]

Table S2 Principal component analysis (PCA) results of surface soil physicochemical properties in the study area

| Principal Component Number | Eigenvalue | Percentage of Variance (%) | Cumulative (%) |
| --- | --- | --- | --- |
| 1 | 3.04 | 60.74 | 60.74 |
| 2 | 1.14 | 22.87 | 83.62 |
| 3 | 0.54 | 10.83 | 94.44 |
| 4 | 0.20 | 4.06 | 98.50 |
| 5 | 0.07 | 1.50 | 100.00 |
