# Supplementary material for: Interaction of soil pH, organic matter, exchangeable acidity, and cation exchange capacity in a managed tea farm
Source: PeerJ. 2025 Nov 24;13:e20341. doi: 10.7717/peerj.20341 (PMC12659706; doi:10.7717/peerj.20341)
Supplement: Supplemental Information 10 [file peerj-13-20341-s010.docx]

Table S3 Factor loading of surface soil physicochemical properties by Principal Component Analysis in the study area

| Variables analyzed | PC1 | PC2 | PC3 |
| --- | --- | --- | --- |
| pH | -0.52 | 0.18 | 0.22 |
| SOM | 0.26 | 0.72 | -0.62 |
| exchangeable H^+^ | 0.48 | 0.30 | 0.53 |
| exchangeable Al^3+^ | 0.54 | -0.01 | 0.33 |
| CEC | -0.37 | 0.61 | 0.42 |
